# Reflection type metasurface designed for high efficiency vectorial field generation

**DOI:** 10.1038/srep29626

**Published:** 2016-07-15

**Authors:** Shiyi Wang, Qiwen Zhan

**Affiliations:** 1 Globalfoundries, 2070 Route 52, Hopewell Junction, NY, 12533, USA; 2 Electro-Optics Graduate Program, University of Dayton, 300 College Park, Dayton, OH, 45469-2951, USA

## Abstract

We propose a reflection type metal-insulator-metal (MIM) metasurface composed of hybrid nano-antennas for comprehensive spatial engineering of the properties of optical fields. The capability of such structure is illustrated in the design of a device that can be used to produce a radially polarized vectorial beam for optical needle field generation. This device consists of uniformly segmented sectors of high efficiency MIM metasurface. With each of the segment sector functioning as a local quarter-wave-plate (QWP), the device is designed to convert circularly polarized incidence into local linear polarization to create an overall radial polarization with corresponding binary phases and extremely high dynamic range amplitude modulation. The capability of such devices enables the generation of nearly arbitrarily complex optical fields that may find broad applications that transcend disciplinary boundaries.

Recently, nano-antenna-based metasurfaces have drawn tremendous attentions due to their extraordinary capabilities for optical manipulation and modulation. By introducing exotic properties, metasurfaces applications have been well investigated in the fields of anomalous reflection and refraction^1^, metasurface QWP^2^,^3^, meta-lenses^4^,^5^, meta-holography^6^,^7^, metasurface deflector^8^, surface plasmon source^9^, vector beam manipulation^10^ and etc. In the quest of miniaturization for photonic device, researchers have realized optical components like polarizers and QWPs on challenging scales through subwavelength periodic arrangement of two-dimensional metallic nano-structures. In particular, by adjusting geometry and periodicity^2^,^3^, metasurface based on interleaved nano-rod antennas or V-antennas has been demonstrated for the implementation of broadband quarter-wave-plates. In addition, the scattering efficiency of reflection type meta-waveplates can be further improved with the use of MIM structure without sacrificing the desired manipulation capability^11^,^12^. Hence, with a circularly polarized illumination, devices that consist of segmented MIM meta-quarter-wave-plates can be designed for high efficiency generation of spatially polarized light with phase and amplitude modulations. These nano-engineered vectorial optical fields find broad potential applications, such as optical needle field (ONF) generation under high numerical aperture (NA) focusing^13^.

The so-called ONF is substantially polarized along the propagation direction with extended depth of focus (DOF) and can be generated through the focusing of filtered radial polarization^14^,^15^,^16^,^17^,^18^. It has attracted many interests as a promising candidate in polarization sensitive orientation imaging^19^, particle manipulation^20^ and so on. Based on the method of reversing electric dipole array radiation, a discrete complex pupil filter has been designed for easier implementation of desired incident field for ONF generation^17^. This complex filter gives rise to spatial polarization, phase as well as amplitude modulations to the input optical illumination. It also motivated a transmission type slot-antenna-based metasurface design for realizing ONF, whose field modulation function has been experimentally verified^18^. However, although this work provides guidance of vectorial optical field engineering, the transmission type structure suffers a very low scattering efficiency. On the other hand, reflection type metasurfaces have become promising candidates for novel optical manipulation owing to their high efficiency and broadband performance^11^,^21^.

In this paper, an MIM hybrid reflection type nano-antenna-based metasurface is demonstrated to illustrate its capability of spatially modulating the phase, amplitude as well as polarization of optical fields with high efficiency. A specific device capable of creating a radially polarized vectorial beam for ONF generation is shown as an example. Functioning as local QWP, MIM metasurface with segmented sectors is designed to convert incident circular polarization into local linear polarization to create an overall radial polarization with corresponding binary phases and desired normalized amplitude modulated from 0.071 to 1^17^. In this design, zones with periodic arrangements of double metallic nano-bars with perpendicular placement, and single nano-bars are carefully engineered to realize different modulation requirements while maintaining π/2 retardation. Besides, MIM disk structures are applied for eliminating undesired reflection through deflection. The overall hybrid metasurface structure integrates several functions within one device, leading to a compact device for vectorial optical field engineering that may enable highly desirable solutions for optical integration.

## Results

### Design of discrete complex reflection modulator for vectorial field generation

**Figure 1:**
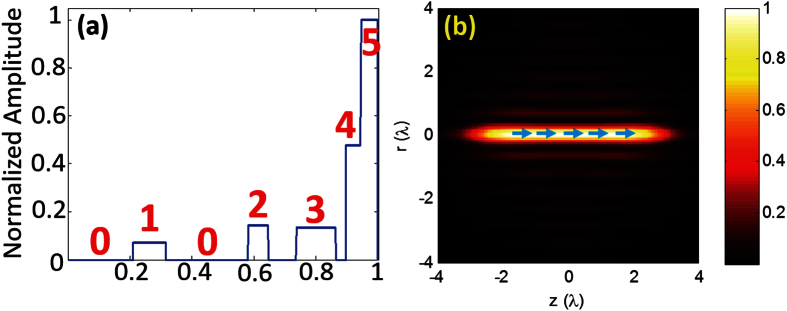
Plots of discrete complex reflection modulator (**a**) Normalized amplitude of discrete reflection filter along radial direction; (**b**) simulated normalized intensity distribution near focus (ONF polarization direction is marked with light blue arrows).

To obtain the desired complex optical field for ONF generation, a method based on reversing the radiation pattern of electric dipole has been developed^17^,^18^. A filter consisting of concentric rings with discrete amplitude and binary phase is designed to obtain extended DOF with uniform axial intensity profile and high purity longitudinal polarization. To simplify the complexity of fabrication and design, we demonstrate a device with five modulation rings for ONF generation with 5λ DOF by following the method reported in^17^ (Fig. 1(a)). The design parameters are summarized in Table 1. After the modulated radial polarization light passing through a high NA lens, a longitudinally polarized light at focus is expected with a flat-top intensity of 5λ DOF (Fig. 1(b)).

### Design of reflection type metasurface based on nano-bars structures

To engineer the complex modulation capabilities with maximal flexibility, a reflection type MIM nano-antenna-based metasurface is chosen due to its high efficiency. With a nano spacer sandwiched between the upper metallic arrays and the bottom mirror-like metal layer, gap plasmon resonance can be exploited to facilitate the reflection light manipulation based on cautious adjustment of structure parameters^22^,^23^. As one of the simplest structures, metal nano-bar is chosen to be the unit cell of the upper antenna layer for the realization of π/2 retardation with high conversion efficiency as demonstrated in^23^. In their work, a broadband reflectance of about 90% can be reached with the support of MIM nano-bar antenna, which can be used to meet the reflection requirement with the highest amount in our design (Ring #5). However, structures with additional degrees of freedom are still needed in order to realize the normalized amplitude modulation ranged from 0.071 to 1.

**Figure 2:**
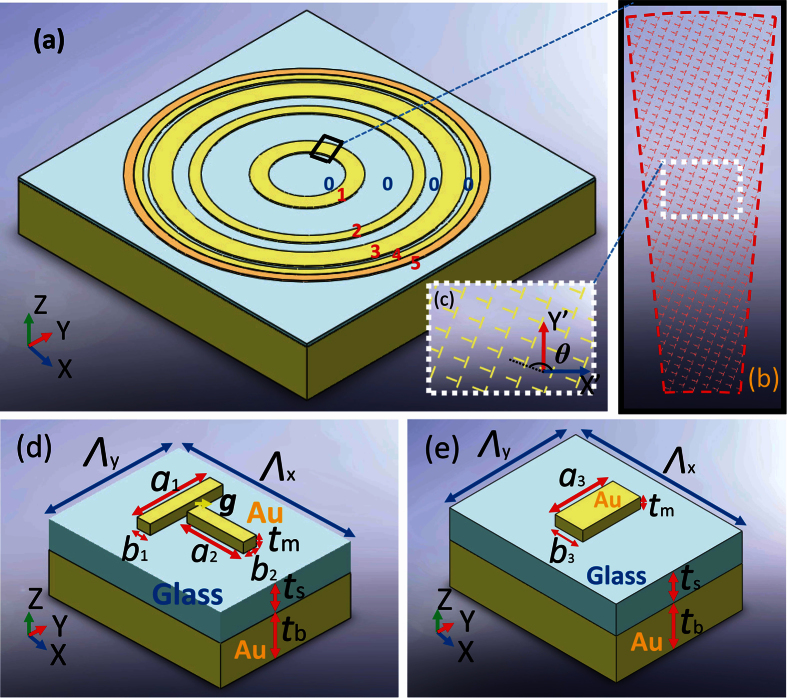
MIM metasurface design (**a**) Overall schematic plot of the reflection type MIM metasurface (Rings #1~#4 with light yellow color are composed of double nano-bars structures, while brownish ring stands for Ring #5 composed of single nano-bars); (**b**) one of the 32 sectors extracted from the first ring; (**c**) zoom-in for double nano-bars array rotated by θ in the local coordinate of the sector; (**d**) schematic plot for orthogonally patterned MIM double nano-bars unit cell; (**e**) MIM single nano-bar unit cell.

To access that modulation range for the other four rings, a metasurface based on interleaved gold nano-bars are employed for the upper layer structure. In our design, the nano-bars are periodically deposited on a glass thin layer with an optically thick gold bottom layer at the bottom, giving rise to an MIM structure or so-called continuous-layer gap plasmon resonators (Fig. 2(d,e))^24^. For each of the unit cells, the resonator basically performs either as a magnetic dipole formed by electric currents flowing through upper metallic bar and bottom layer or an electric dipole depending on resonance conditions. With proper choice of unit cell dimension, the desired gap plasmon resonance can be controlled, resulting in minimum reflection with most of energy being stored within the glass spacer region. From this point, the structures enable the functions either as on-resonance perfect absorbers or off-resonance reflective components handled through adjustment of the geometry. Furthermore, if two orthogonal arrays of nano-bars are interleaved with each other, a desired reflection phase difference can be engineered by keeping one array of nano-bars above resonance and the other below resonance. Particularly, by perpendicularly positioning two arrays of nano-bars with a proper nano spacing for coupling purposes, the π/2 phase retardation can be reached with each of the nano-bars corresponding to orthogonal polarizations^3^,^25^. Hence, behaving as a local QWP through balancing the phase retardation, the metasurface can generate a radially polarized modulated field if a circular polarization light is applied (Fig. 2(a)). With considerable degrees of freedom, these interleaved nano-bar MIM structures are expected to cover our amplitude modulation range while maintaining the phase retardation to be π/2.

Extracted from a rectangular lattice with *Λ*_x_ by *Λ*_y_ subwavelength periods, the unit cell of orthogonally patterned nano-bar MIM structures is displayed in Fig. 2(d). A glass spacer layer with thickness *t*_s_ is sandwiched between an antenna layer composed of gold nano-bars array and a bottom reflection layer. The bottom layer thickness *t*_b_ is chosen to be over 80 nm, which can be considered as a perfect reflector at our working wavelength 1064 nm. The two nano-bars are placed perpendicularly along their corresponding axes respectively with a separation gap of *g*, giving rise to an overall symmetric “T” shape. Through balancing the effective coupling distance and fabrication capability, the separation gap *g* is fixed to be 10 nm. The lengths and widths of nano-bars are expressed by *a*_*i*_ and *b*_*i*_ (*i* = 1, 2) as shown in the plot, which requires systematic exploration for resonance in pursuit of the desired reflection field. Similarly, in Fig. 2(e), the single nano-bar (*a*_3_ × *b*_3_) is deposited on the same glass layer.

Consisting of the corresponding nano-antenna arrays, each ring is split into 32 uniform centrosymmetric sectors as shown in Fig. 2(a). In one specific ring, each of the 32 sectors has the same configurations of periodicity and geometry for the nano-antenna. Here, the scattered field is investigated in the local coordinates (X’, Y’) for the sector region, where they are actually the azimuthal and radial directions respectively under the laboratory coordinates. In order to create an overall radial polarization output, the two-dimensional antenna array is required to rotate by an amount of *θ* (Fig. 2(c)) to align local output linear polarization to local Y’ axis. With proper structure design, the retardation, defined as “Δ*ϕ* = (*ϕ*_y_ − *ϕ*_x_)”, can be fixed as ±π/2, and thus a QWP function is realized. Under normal incident circular polarization illumination, the output Jones vector *J*_out_ can be expressed as (for both ±π/2 cases),


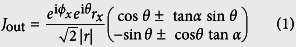


Here, the amplitude of reflectance |*r*| is expressed by *r*_x_ and *r*_y_ as 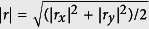; “*α*” is the angle intersected between the X axis and the output linear polarization direction with “tanα = *r*_y_/*r*_x_”. Therefore, when Δ*ϕ* = π/2, let *θ* = π/2 + *α*, then:


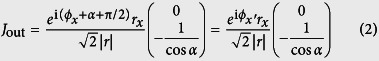


where 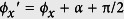. On the other hand, when Δ*ϕ* = −π/2, let *θ* = π/2 − *α*, then:


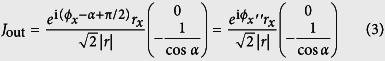


where 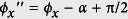. *ϕ*_x_′ and *ϕ*_x_′′ are modulated reflection phase along X axis, which are actually the binary phases for different rings given in Table 1.

**Figure 3:**
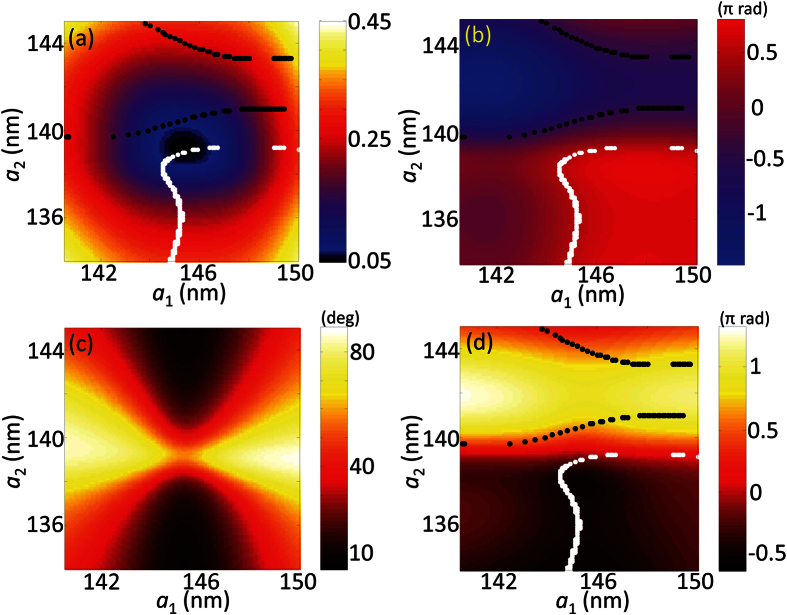
2D mapping plots for MIM metasurface Ring #1 (Λ_x_ = 330 nm, Λ_y_ = 250 nm, b_1_ = b_2_ = 25 nm) (**a**) the amplitude of reflection |*r*|; (**b**) phase retardation Δ*ϕ*; (**c**) output linear polarization angle α; (**d**) modulated reflection phase *ϕ*_x_’.

**Figure 4:**
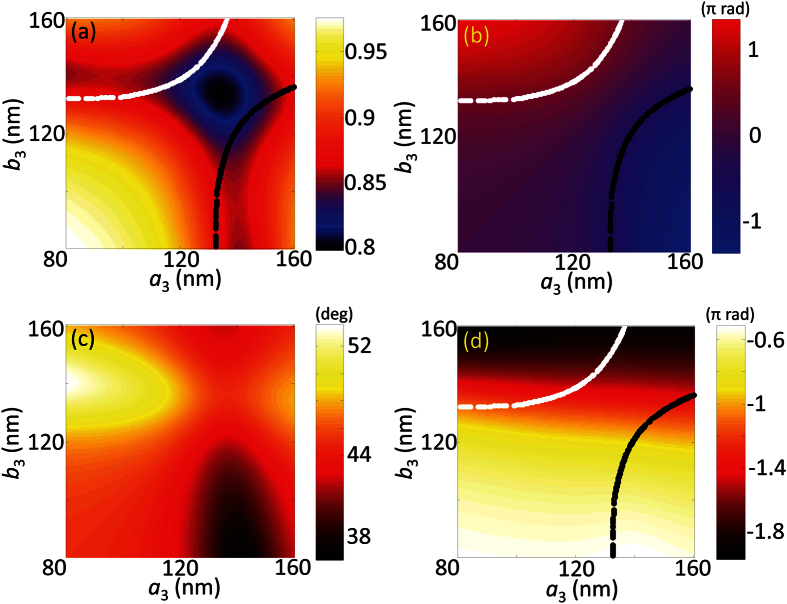
2D mapping plots for MIM metasurface Ring #5 (*Λ*_x_ = *Λ*_y_ = 190 nm) (**a**) the reflection amplitude |r|; (**b**) phase retardation Δ*ϕ*; (**c**) output linear polarization angle α; (**d**) modulated reflection phase *ϕ*_x_”. (The dotted lines represent the retardation contour for −π/2 (black) and π/2 (white) respectively).

To meet the modulation requirement, we systematically investigate the geometric parameters for the nano structures. In this design, 3D modelling and numerical simulation for unit cell is accomplished based on finite element method (FEM, COMSOL Multiphysics^TR^) with the permittivity of materials from Palik’s^26^. Under periodic boundary conditions, the double nano-bar antenna is illuminated by normal incident light with X and Y polarization respectively. Then, the complex reflection field is collected at the input port for further analysis. Firstly, to find the candidate structures, the thicknesses of the layers require optimization for minimum reflection, while fixing the other system parameters as constants (*Λ*_x_ = *Λ*_y_ = 260 nm, *b*_1_ = *b*_2_ = 25 nm, *a*_1_ = 145 nm, *a*_2_ = 135 nm, initial parameters are chosen based on fabrication capability). In this case, the MIM structure behaves similarly to the well-known metamaterial perfect absorber^27^,^28^, whose resonance condition can be found based on the transmission line theory. This theory is borrowed from LC circuit design that can realize lowest reflection by satisfying the impedance matching to free space. By adjusting the thicknesses of nano-antenna *t*_m_ and spacer *t*_s_, the minimum reflection amplitude |*r*| of 0.166 is obtained when *t*_m_ = 20 nm and *t*_s_ = 55 nm, which are within the fabrication capability of available tools. From this configuration, further optimization is implemented through the adjustment of unit cell period. For Ring #1, an asymmetric rectangular lattice (*Λ*_x_ = 330 nm, *Λ*_y_ = 250 nm) is used to reach the resonance conditions of gap plasmon for both directions respectively. Under on-resonance state, a reflection amplitude |*r*| as low as 0.056 can be obtained based on proper choice of nano-bars dimension as shown in Fig. 3(a). If we slightly change the current parameters, one of the nano-bars can be shifted above resonance, while the other below resonance. This gives rise to the possibility of flexible control for phase difference. With appropriate amount of shift, two retardation contours for −π/2 (black) and π/2 (white) can be found as represented by the dotted lines. By searching parametric combinations along these contours in both Fig. 3(a,d), the requirements for both amplitude and phase can be satisfied while maintaining the QWP function. After cautious considerations, a double nano-bar unit cell with *a*_1_ = 146.4 nm, *a*_2_ = 139.2 nm, *b*_1_ = *b*_2_ = 25 nm is selected to achieve a reflection amplitude |*r*| of 0.0585 and a modified reflectance phase *ϕ*_x_′ of 0 rad at the same time. Also, by following Fig. 3(c), the rectangular lattice needs a counterclockwise rotation with the amount *θ* of 159.1 degrees (*θ* = π/2 + *α*) to align the output linear polarization to the radial direction under the laboratory coordinates. Similarly, parameters for the double bars nano-antenna in Ring #2, #3 and #4 can be found with the same methodology. On the other hand, in the case of Ring #5, a single nano-bar MIM unit cell is chosen due to its relatively high reflection and straightforward geometry (Fig. 2(e)). Similarly, by fixing its lattice to be square array of 190 nm periodicity as illustrated in Fig. 4, the amplitude reflection |*r*| can be reached as high as 0.826 with a modified reflection phase *ϕ*_x_′′ of π rad. All of the geometric configurations for the five segmented QWP rings that can completely satisfy the modulation requirements are summarized in Table 2.

### Design of MIM disk structures

**Figure 5:**
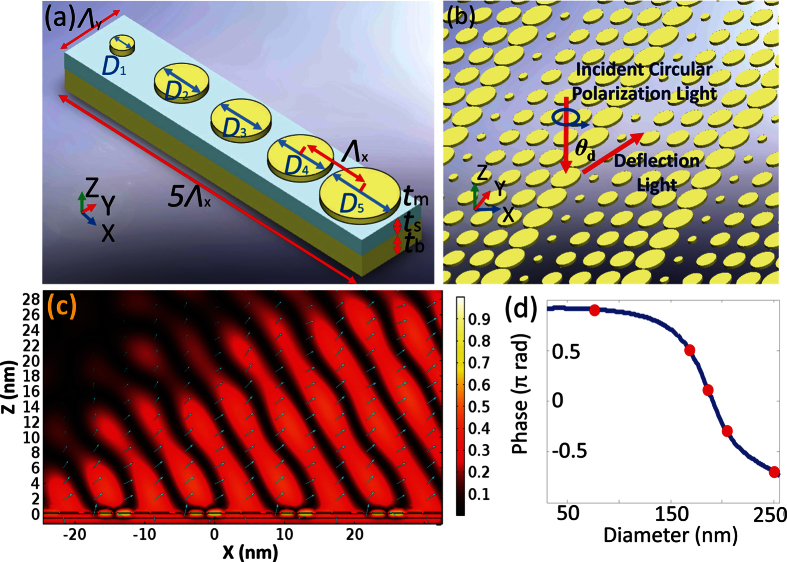
MIM disk structure design (**a**) MIM disk structures unit cell; (**b**) deflection behavior of MIM disk array; (**c**) scattered electric field distribution for 5 super-periods of unit cell with Y polarized incident light (the relative power flow is marked with light blue arrows); (**d**) disk diameter vs. reflection phase (the chosen diameters are marked with red dots).

To eliminate the undesired reflection light within Regions #0 (Fig. 2(a)), an MIM disk metasurface with the same thickness is applied to deflect the incident circular polarization light off normal direction. The disk structure is chosen to handle the circular polarization input considering its polarization-independent shape. With the square period fixed as 260 nm for each of sub-units, the reflection phases of different disk sizes are simulated and a curve of disk diameter vs. reflection phase is shown in Fig. 5(d). Five gradual phases with a constant step of 0.4π rad are extracted to cover a full wave of 1064 nm (Table 3). By placing these 5 sub-units of MIM disk structures along the X-axis, a super-periodic array (5*Λ*_x_ × *Λ*_y_) is obtained that performs the deflection function through anomalous reflection (Fig. 5(a,b)). The deflection angle *θ*_d_ is calculated as 54.9 degree^1^.

Furthermore, without loss of generality, we simulated the deflection performance with Y polarized incident light for the narrowest part in this design (the region between Ring #3 and #4), which contains merely 5 super-periods along X axis as shown in Fig. 5(c). The light blue arrows represent the relative power flow given by the scattered field, whose lengths illustrate the power flow amplitude in logarithmic scale. Here, the simulated deflection angle is found to be 54.6 degree, which agrees very well with the theoretically predicted value. Apparently, higher deflection efficiency is expected if more super-periods are added to the structure. To check the completeness of our model, the same structure is illuminated with X polarized incident light resulting in *θ*_d_ of 55.4 degree. Hence, with engineered super-periodic structures filled in the gaps between those rings, a near-zero reflection in normal direction is realized. This completes a hybrid MIM metasurface design with proper choice of three different nano-antennas that can be used for ONF generation at near IR wavelength.

## Discussion

In conclusions, a hybrid reflection type MIM metasurface of nano-antennas at near IR wavelength is designed, modelled and numerically proved for vectorial light engineering. Functioning as a segmented QWPs, it converts incident circular polarization to radially polarized complex field with modulated amplitude, phase and specific spatial polarization. The gap plasmon resonance derived from the structures is manipulated by adjusting the antenna dimensions in pursuit of desired optical performance. Using nano-antenna arrays, the modulated field can cover a normalized amplitude range from 0.071 to 1 with binary phase control. As an example, a device design that is capable of creating complex field for ONF generation with a flat-top DOF of 5λ is demonstrated. The reflection type MIM metasurface offers promising possibilities for a new class of compact optical components for complex vectorial light generation and manipulation based on nano-scaled structures.

## Methods

### Simulation method

The full-wave simulations of the characteristics of metasurface unit structures are performed using the radio frequency module of the commercial software COMSOL. The unit MIM structure shown in Figs 2(d,e) and 5(a) are surrounded by periodic boundaries conditions, which are applied to simulate the overall periodicity. The scattered electric field distribution is calculated by the post-processing available in COMSOL.

### Theoretical derivation of output scattered field in Jones vector form

Under normal incident circular polarization illumination, the output Jones vector *J*_out_ can be derived as,


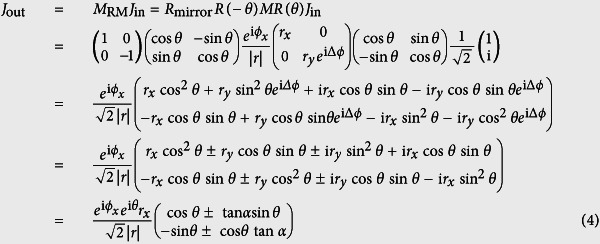


Here, *J*_in_ is the normalized circular polarization input; *M*_RM_ is the Jones matrix representation of reflection metasurface; *R*_mirror_ is the reflection factor; *R*(*θ*) is the rotation matrix with the angular amount of *θ*.

### Calculation of deflection angle *θ*_d_


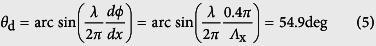


## Additional Information

**How to cite this article**: Wang, S. and Zhan, Q. Reflection type metasurface designed for high efficiency vectorial field generation. *Sci. Rep.* **6**, 29626; doi: 10.1038/srep29626 (2016).

## Figures and Tables

**Table 1:** Design parameters for discrete complex reflection metasurface design in polar coordinate.

**Ring #**	**1**	**2**	**3**	**4**	**5**
|*r*|_norm_	0.071	0.145	0.133	0.478	1
*ϕ*(rad)	0	π	0	π	π
*w*_norm_	0.105	0.065	0.13	0.05	0.055
*r*_c_	0.263	0.613	0.800	0.920	0.973

(|*r*|_norm_: normalized amplitude; *ϕ*: phase; *w*_norm_: normalized ring width; *r*_c_: ring center position).

**Table 2:** MIM metasurface structures parametric configurations.

**Ring #**	**1**	**2**	**3**	**4**	**5**
|*r*|	0.0585	0.12	0.11	0.395	0.826
|*r*|_norm_	0.071	0.145	0.133	0.478	1
*φ*(rad)	0	π	0	π	π
(*a*_1_, *a*_2_)(*b*_1_, *b*_2_)(nm)	(146.4, 139.2)(25, 25)	(134.9, 127.4)(20, 20)	(145.3, 141.8)(25, 25)	(154.8, 153.0)(45, 45)	(*a*_3_, *b*_3_)= (141.0, 123.3)
(*Λ*_x_, *Λ*_y_) (nm)	(330, 250)	(240, 230)	(330, 260)	(330, 250)	(190, 190)
*α* (deg)	69.1	3.7	30.0	50.4	43.2
*θ* (deg)	159.1	86.3	60.0	140.4	46.8

**Table 3:** Reflection phases of the chosen disk diameters (*D*: diameter in nm; *φ*_D_: phase in π rad).

	**1**	**2**	**3**	**4**	**5**
*D*	76	168	186	204	250
*φ*_D_	0.91	0.51	0.11	−0.29	−0.69
